# Experiments support an improved model for particle transport in fluidized beds

**DOI:** 10.1038/s41598-017-10597-3

**Published:** 2017-08-31

**Authors:** Huili Zhang, Weibin Kong, Tianwei Tan, Flamant Gilles, Jan Baeyens

**Affiliations:** 1 Beijing University of Chemical Technology, School of Life Science and Technology, Beijing, China; 2 Promes-CNRS, Centre National de Recherche Scientifique, Font Romeu, France; 3 European Powder and Process Technology, 3120 Tremelo, Belgium

## Abstract

The upwards flow of particles in an Upflow Bubbling Fluidized Bed (UBFB) is studied experimentally and modelled from pressure drop considerations and energy loss equations. For Geldart group A powders tested, the upward solid flux, *G*_*s*_, in the tube can be expressed in terms of the applied superficial gas velocity, the free fall (terminal) velocity of the particles during their hindered settling, *KU*_*t*_, the pressure exerted at the base of the conveyor tube, and the tube length. The model expression ${{\boldsymbol{G}}}_{{\boldsymbol{s}}}=\frac{{\boldsymbol{\Delta }}{\boldsymbol{P}}}{({U}_{{\boldsymbol{g}}}-{\boldsymbol{K}}{{\boldsymbol{U}}}_{{\boldsymbol{t}}}){\boldsymbol{+}}\frac{{{\boldsymbol{K}}}^{{\boldsymbol{2}}}{\boldsymbol{g}}{\boldsymbol{L}}}{({{\boldsymbol{U}}}_{{\boldsymbol{g}}}-{\boldsymbol{K}}{{\boldsymbol{U}}}_{{\boldsymbol{t}}})}}$ can be used for design purposes, with *K*, the correction factor for hindered settling of the particles, approximately equal to 0.1 at high *G*_*s*_-values, but a function of the solids fraction in the upward conveying. The energy efficiency of the system increases with increasing U and G_s_. The model equation was tentatively applied to predict the effects of particle size, tube length and operation in Circulating Fluidized Bed mode. It is demonstrated that the UBFB is an efficient and flexible way of transporting particles upwards, with limited particle attrition or tube erosion due to the low gas velocity applied.

## Introduction

## Powder properties and common conveying systems

Gas fluidization is applicable to different powders, classified by Geldart^1^ according to C (cohesive), A (aeratable), B (bubbling) and D (coarse) particles. The transition between the different powder classes can be expressed in terms of surface/volume mean diameter of powders (*d*_*sv*_), absolute particle density (*ρ*_*s*_) and density of the fluidization gas (*ρ*_*g*_), by appropriate equations^2^.

Group A powders are widely used in fluidized bed catalytic reactors. Their recent application in Upflow Bubbling Fluidized Bed (UBFB) solar receivers opens new perspectives^3, 4^. Cracking catalyst and fine sand (<90 μm) are typical group A powders. Their beds expand significantly at superficial gas velocities (U or U_g_) between minimum fluidization gas velocity (*U*_*mf*_) and minimum bubbling gas velocity (*U*_*mb*_). For gas velocities above *U*_*mb*_, bubbles form and disrupt the meta-stable expanded bed structure: the bed height (H) is reduced to bed height at U_mf_ (*H*_*mf*_) with a bed voidage (*ε*) between the voidage of the bed at U_mf_ (*ε*_*mf*_) and the voidage of the bed at U_mb_ (*ε*_*mb*_). A further increasing gas velocity will produce a net increase in bed expansion^1^ due to bubbling. Group A powders are easily circulated around fluidized and pneumatic conveying loops. Bubbles induce a gross circulation of the powders, similar to gulf streaming in liquids^5^, producing considerable particle mixing. At higher gas velocities, bubbles split and recoalesce frequently, resulting in a maximum stable bubble size if the diameter of the bed is large enough to avoid slugging^2^. The bubble size is not affected by the *d*_*sv*_ of group A powders. The rise velocities of small bubbles (<5 cm) are around 0.3 to 0.4 m/s, regardless of the bubble size, suggesting that the particle gulf streaming controls the rise velocity^6^. At high superficial gas velocity, bubbles will mostly transform into wall slugs^2^. Based upon previous research and a literature review for group A particles^4, 7–16^, the conveying of particles can be performed in different ways, as illustrated and assessed in Tables S1 and S2. Previous research on the UBFB and Circulating Fluidized Bed (CFB) is detailed in Table S3.

## The novel applications of the UBFB conveyer concept

A first application focuses on Concentrated Solar Power plants (CSP). CSP plants are dynamic, flexible, and offer an adaptable power output when a Thermal Energy Storage (TES) system is included^17, 18^. CSP plants operate mainly with water/steam, thermal oils or molten salt as primary heat carrier, thus limiting the maximum operating temperature^4^. To make CSP technology more competitive, current research aims to increase operating temperatures of the heat carrier in order to increase the efficiency of the power cycle, to reduce receiver losses by higher fluxes, to reduce the size of the heliostat field, and to reduce the capacity of the cold and hot storage.

**Figure 1:**
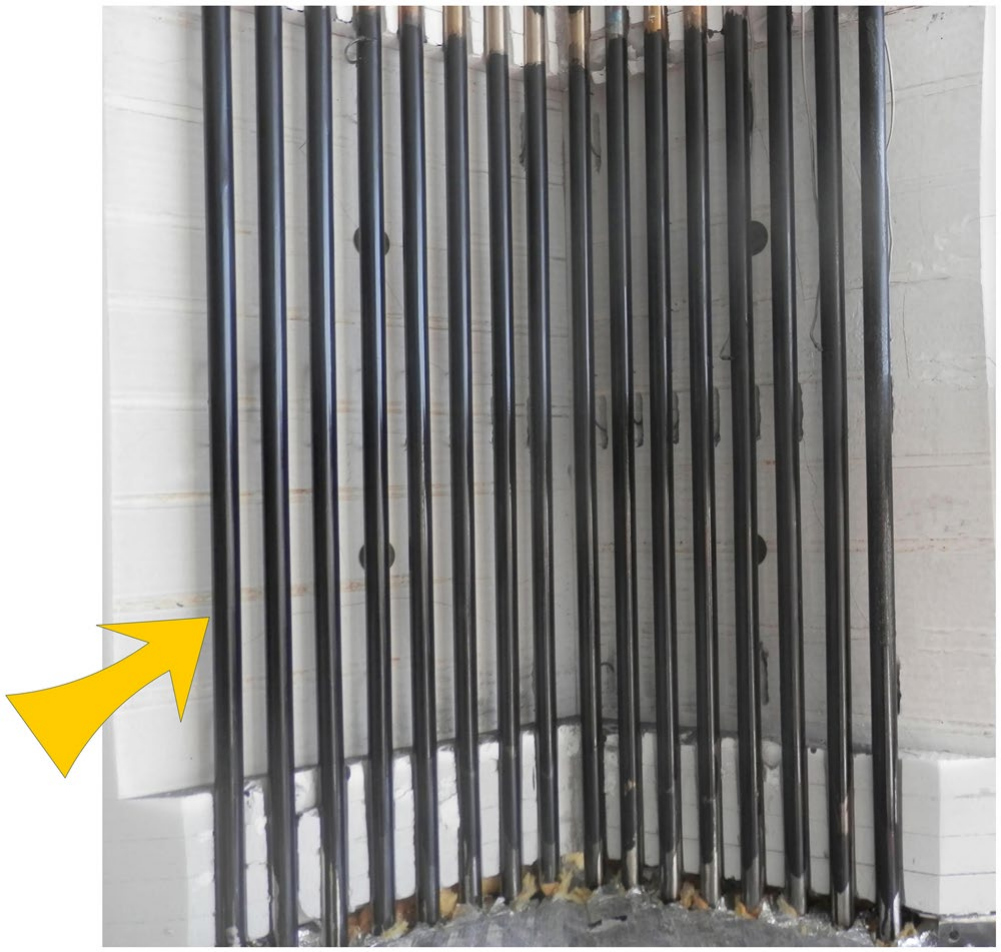
3D view of the pilot multi-tube solar receiver (Credit: PROMES-CNRS), with solar cavity, refractory lined; and 16 parallel UBFB receiver tubes;  concentrated solar irradiance flux.

Using particles as heat transfer medium meets these targets since they are not subject to the upper (decomposition) and lower (solidification) temperature limitations of molten salts, and provide a good thermal capacity and a low cost heat transfer and storage material^4^. Direct and indirect particle receivers are being developed. Indirect particle-in-tube receivers can efficiently use an entire elliptical field, and can be scaled-up more readily. Concepts of CNRS^19, 20^ and NREL^21, 22^ have been published. The present work considers the design and operation of the CNRS concept, referred to as particle-in-tube or Upflow Bubbling Fluidized Bed (UBFB) technology. The on-sun testing of a single tube (36 mm Internal Diameter, I.D., 0.5 m long) and of a 150 kW pilot module consisting of 16 parallel tubes of 29.7 mm I.D., each 1 m long, has been reported previously and is illustrated in Fig. 1 ^3, 4^. Group A powders are used and fluidized at superficial gas velocities of ~0.03 to ~0.20 m s^−1^.

A currently ongoing scale-up to a multi-MW capacity will involve the use of the parallel tube-concept, with 40 parallel tubes of 0.05 m I.D. tubes and of 3 m length to efficiently make use of the concentrated heliostat beams and provide sufficient heat capture surface area^13^. Contrary to the bubbling hydrodynamics in short tubes, the gas-solid hydrodynamics in the UBFB was recently proven to be of combined bubbling and slugging nature, and strongly affected by the geometry of the tubes, with special emphasis on their height, since the common unrestrained bubbling is transformed into slugging when the bubble size is a 50 to 60% fraction of the I.D. of the tube. The effect of the tube length is hence of paramount importance^2^.

A second potential application^23^ aims to manage plasma flows which could be used in nuclear fusion reactions. Tungsten-coated SiC particles are dropped through the plasma as it leaves the fusion zone to reduce the plasma energy prior to absorbing the remainder of the plasma energy through diverter plates. Particles are then cooled and returned to the top of the reactor within a closed environment^23^. Conveying in a dense regime at low gas velocities is recommended to limit the flow rate of carrier gas and to reduce particle attrition and conveying line erosion (thus eliminating the application of common pneumatic conveying or CFB).

Finally, the use of a fluidized bed as feeding device of the UBFB conveyor enables the pre-mixing of dissimilar materials within specific wt% ranges^24^, as e.g. applicable in UO_2_-HF fluorination for nuclear fuel reprocessing with an Al_2_O_3_ bulk bed, or in co-conveying cohesive group C particles.

## Objectives of the study

The research aims to develop a reliable method for the design and scale-up of the UBFB conveying systems, based on experiments in the CNRS concept. Since it was recently demonstrated and proven that long UBFB columns of small I.D. operate in both bubbling and slugging fluidization mode^2^, the currently developed analytical or numerical methods are inadequate, due to the uncertainties of some of the parameters, such as the extent of freely bubbling, slug characteristics, the stress state of the solid plugs in between successive slugs, and the considerable gulf streaming of the particles near the wall. Moreover, Most pneumatic conveying and CFB semi-empirical models are focused on the operation from dilute to dense mode, without feeding fluidized bed, but with the solids feed into the conveying pipe either in an in-line pre-mixing chamber, or using a side L-valve for CFBs respectively.

The development of a more generalized method is required to combine the relevant characteristics of particle, gas, conveying pipe and pressure drop (Δ*P*). This is further developed in the present paper from initial experimental results and theoretical considerations.

## Experimental

### Equipment and powders used

**Figure 2:**
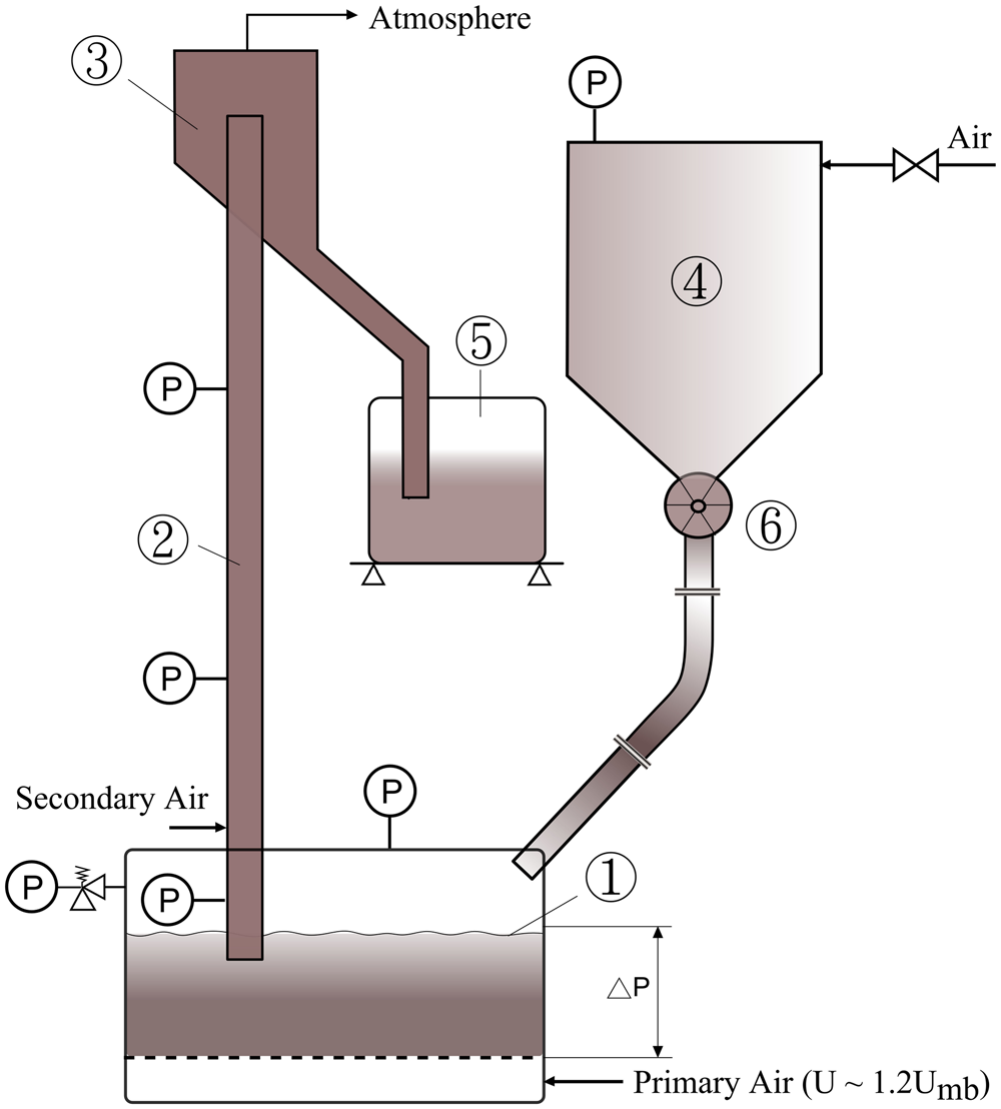
Experimental layout, ➀ Dispenser FB with pressure setting and relief valve; ➁ upflow tube; ➂ disengagement chamber; ➃ pressurized feed of dispenser; ➄ weighing collector of powder after conveying; ➅ calibrated rotary value.

The experimental layout is illustrated in Fig. 2. Small diameter tubes were used of 29.6mm I.D. for a tube length of 2.1 m, and a 50 mm I.D. of 1 m length, followed by a disengagement section of 0.3 m height. The columns were earthed. The fluidized bottom bed (called dispenser) feeding the tubes was fitted with a micro-porous metal distributor. The bed height in the dispenser was maintained at 0.25 m. The conveying tube inlet is located 5 cm below the bed height. The experimental rigs have been previously described in detail by various authors^4, 13, 19, 25, 26^.

The valve-controlled supply of dry, oil-free compressed air was metered by a digital flow meter, calibrated using both a wet gas meter and by the water displacement method. All experiments were carried out with air at ambient temperature. Both SiC and cristobalite silica were used, with properties as given in Table 1. SiC was used in the 29.6 mm I.D. tube, cristobalite was used in the 50mm I.D. tube. The dispenser was fluidized at ~1.2 U_mb_. Secondary air increased the total superficial air velocity to between 0.02 and 0.16 m/s.

**Table 1:** Particle properties.

Powder	*d*_*sv*_ (μm)	σ (μm)	(*ρ*_*s*_) (kg m^−3^)	*(ρ*_*B*_ *)* (kg m^−3^)	*U*_*mf*_, *U*_*mb*_ (cm/s)	*ψ*	*U*_*t*_ (m/s)
SiC	64	12.1	3210	1583	0.55/0.80	0.65–0.7	0.309
Cristobalite	58	14.0	2340	1155	0.34/0.53	0.7–0.75	0.201

Particle sizes and the size distribution were measured by Malvern laser diffractometer (Mastersizer 2000). *U*_*mf*_ and *U*_*mb*_ were determined experimentally, and are in agreement with predictions by Wu and Baeyens^27^ and Geldart and Abrahamsen^28^, respectively. Particle shapes were determined by OPTEC DV 320 microscope imaging. About 30 particles of both SiC and cristobalite were viewed. The average particle sphericity (*ψ*) was calculated according to Cavarretta *et al*.^29^ and Cho *et al*.^30^ and taken as the ratio of the perimeters of the maximum inscribed circles and minimum circumscribed circles to the particles. The bulk density of particles (*ρ*_*B*_) is the tapped bed density. The terminal (free falling) velocity of particle (*U*_*t*_) was calculated according to the method described by Geldart^31^.

The particle sphericity affects *U*_*mf*_ and *U*_*t*_, commonly decreasing as ψ increases, as demonstrated by Geldart^31^ and Kunii-Levenspiel^32^. σ is standard deviation of the particle size distribution.

Particles within the fluidized bed dispenser move upward in the conveying tube by (i) both the pressure difference imposed between the particle suspension at the tube bottom and the atmospheric disengagement chamber at the tube top, and (ii) by the drag force on the particles from the total of primary and secondary air flows. Pressure and differential pressure probes were installed at several positions.

## Experimental Results

For each value of the total superficial air velocity (as sum of primary and secondary air), the solids flow rate was determined by continuously weighing the collected powders during steady-state operations of 5 minutes each. The solid flux was determined per unit time and unit cross sectional area of the respective tubes. Pressure drops were continuously monitored and showed great stability, with variations of less than ±2%. The pressure gradients were determined as the pressure difference between the bottom of the tube, and the atmospheric exhaust of the disengagement chamber, and expressed per meter of corresponding tube length. These gradients for the SiC experiment were 12500 Pa/m at 0.03 m/s and 11500 Pa/m at 0.15 m/s. For cristobalite, they were 8800 Pa/m at 0.02 m/s and 8400 Pa/m at 0.15 m/s. These decreased pressure gradients with increasing air flow rate are due to a higher bed voidage^2^, and hence lower solid fraction (α_p_), since (1-ε) = α_p_ = $\frac{{\rm{\Delta }}P}{L{\rho }_{s}g}$. From the ΔP measurements, α_p_ decreased from 0.39 to 0.36 for SiC, whereas for cristobalite, the values ranged from 0.38 to 0.36. Within the range of operating air velocities, an average value for both powders of α_p_~0.37 appears acceptable. Higher superficial velocities and/or lower solid flux at the set air velocities will however further reduce α_p_.

**Figure 3:**
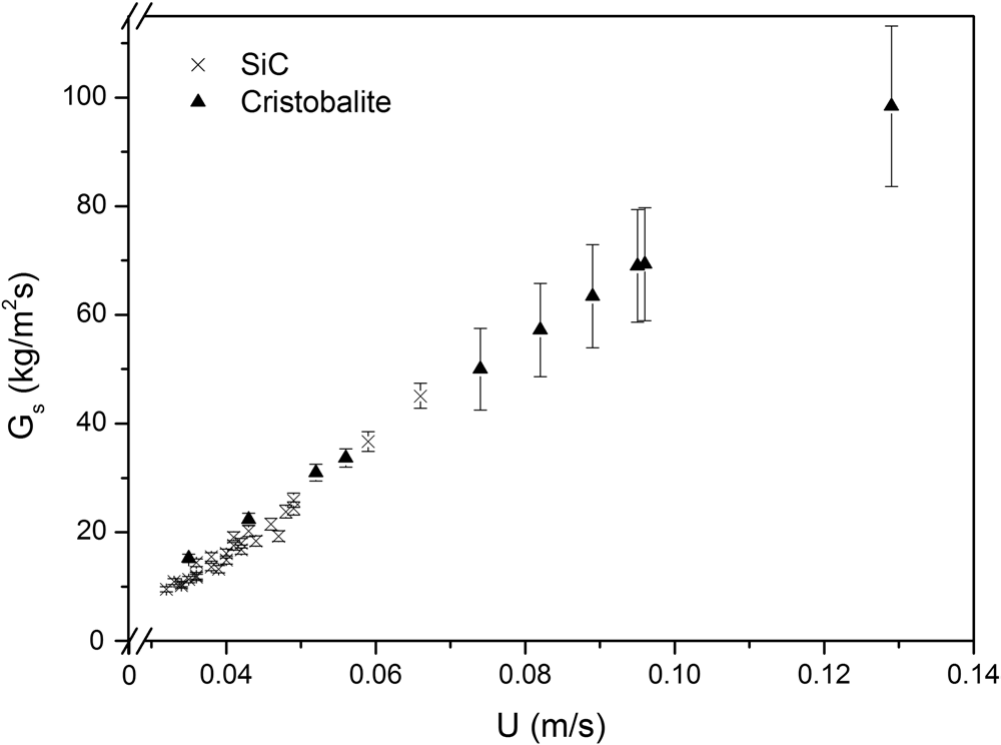
Experimental upward solid flux versus total superficial air velocity.

Figure 3 shows the experimental results for both SiC and cristobalite for a dispenser pressurization at 26500 Pa (SiC) and 9000 Pa (cristobalite), respectively in a 2.1 m tube (SiC) or 1 m tube (cristobalite). The measurement accuracy varies from <+/− 1% at low *U*-values to max. + /− 11% at 0.13 m/s.

Experimental results will be further analyzed qualitatively and quantitatively.

### Modelling approach

#### Pressure balance considerations in a continuous conveying loop

**Figure 4:**
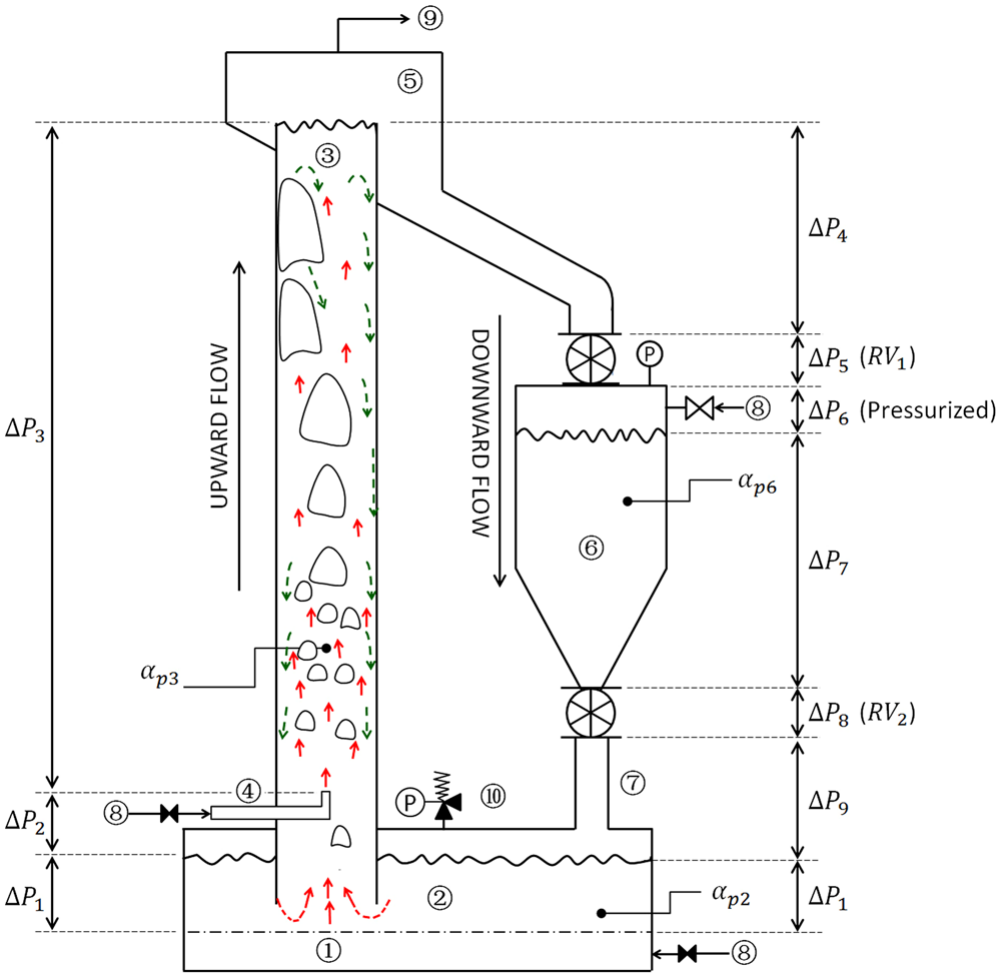
Particle movement and pressure balance in the UBFB,  bubble and *G*_*s*_-driven particle upflow  gross particle downflow near the wall; ➀ windbox or plenum chamber; ➁ fluidized bed dispenser (U~1.2U_mb_) with ➉ pressure relief valve; ➂ up-flow fluidized bed; ➃ secondary air injector; ➄ disengagement chamber; ➅ pressurized hopper; ➆ downcomer; ➇ compressed air; ➈ to fines filtration. RV: rotary valve.

The layout of a continuous UBFB loop, as used in e.g. solar receivers, is represented in Fig. 4. The dispenser bed is operated at a fluidization velocity close to U_mb_ (~1.2 to 1.5 U_mb_). The major part of the conveying gas is injected at the bottom of the tube (about 10 cm above the bottom location of the upward conveying tube). In addition,, different factors should moreover be considered in the loop. The solid fraction, *α*_*p*_, in the upward part of the circuit (the riser) is lower than *α*_*p*_ in the downcomer parts, being 0.35–0.40 and 0.45–0.50 respectively. Rotary valves RV1 and RV2 not only create an additional pressure drop, but also control the bed level of ➅ and the rate of solid circulation, respectively. The pressurization of hopper ➅, indicated as Δ*P*_6_, and subsequent downer parts of the circuit adds to the pressure balance over the whole system.

To operate the loop in a stable flow mode, driving downflow pressures (including the pressurization of hopper ➅) should exceed pressure drops of the upflow branch including acceleration and friction losses, Δ*P*_*f*_. This is represented as follows:

1 $$\sum _{4}^{9}{\rm{\Delta }}{P}_{i}+{\rm{\Delta }}{P}_{1}\ge \sum _{2}^{3}{\rm{\Delta }}{P}_{i}+{\rm{\Delta }}{P}_{f}$$

The dispenser bed exerts a pressure on the fluidized solids proportional to the bulk density and bed height above the inlet of the vertical tube. If this effective bed depth is H_DB_, the pressure drop exerted is

2 $${\rm{\Delta }}{P}_{DB}={\rho }_{s}(1-\varepsilon ){H}_{DB}\,g$$

Since the feeding loop is moreover pressurized at an external pressure Δ*P*_*ext*_, the total driving force is

3 $${\rm{\Delta }}P={\rm{\Delta }}{P}_{ext}+{\rm{\Delta }}{P}_{DB}$$

The available pressure is dissipated in 4 ways:

(i) the energy loss, due to the gas friction in the tube: the related pressure drop is normally very low (<<1% of the total ΔP only)^33^ and can be neglected;

(ii) the energy loss, due to the acceleration of the particles to conveying velocity;

(iii) the energy loss, due to the friction of conveyed particles on the tube wall;

(iv) the energy loss, due to the weight of the particles in the column.

These energy losses can be expressed in terms of the ΔP generated.

The energy loss, ΔP_acc_, to accelerate the particles from zero to the transport velocity, U_s_, is expressed as Eq. (4):

4 $${\rm{\Delta }}{P}_{acc}={\int }_{0}^{{U}_{s}}{G}_{s}d{U}_{s}={G}_{s}{U}_{s}$$

The pressure drop exerted by the solids weight in the tube is given by

5 $${\rm{\Delta }}{P}_{t}=gL{\rho }_{s}{\alpha }_{p}$$

According to the “Fanning” definition, the friction loss is the product of a friction coefficient and friction surface, and an energy per unit volume. The pressure drop due to particle-to-wall friction can be expressed in terms of the particle slip velocity, Δ*U* = *U*_*g*_ − *U*_*s*_, as^33, 34^

6 $${\rm{\Delta }}{P}_{sf}=\frac{3{C}_{D}{\rho }_{g}L}{\,4{d}_{sv}{\rho }_{s}}({\rho }_{s}{\alpha }_{p}){({\rm{\Delta }}U)}^{2}$$

Combining equations (3), (4), (5) and (6), with *ρ*_*s*_ *α*_*p*_ = *G*_*s*_/*U*_*s*_, results in

7 $${\rm{\Delta }}P={G}_{s}{U}_{s}+gL{\rho }_{s}{\alpha }_{p}+\frac{3{C}_{D}{\rho }_{g}L}{4{d}_{sv}{\rho }_{s}}\frac{{G}_{s}}{{U}_{s}}{({\rm{\Delta }}U)}^{2}$$

Normally the slip velocity in lean systems, is expressed as Δ*U* = *U*_*g*_ − *U*_*s*_ = *U*_*t*_^35, 36^.

Since the particles in the tube are hindered by their surrounding dense particle phase, the terminal velocity cannot be reached, and the effective “terminal” velocity will be a fraction of *U*_*t*_ only, as expressed in Eq (8).

8a $${\rm{\Delta }}U={U}_{g}-{U}_{s}=K{U}_{t}$$

8b $${U}_{s}={U}_{g}-K{U}_{t}$$

*K* is supposed to be a function of the bed voidage (*K* = *ε* ^*n*^), with exponent n cited in literature as 4.65 further to the assumed analogy of gas-solid and liquid-solid fluidization and sedimentation^37^, later^38, 39^ corrected to minimum 3.89, with high values obtained when particle sizes are below ~60 µm^40^. As will be shown in section 5, the experimental results favour the use of 4.65, which was hence accepted in Eq. (9):

9 $$K={\varepsilon }^{4.65}={(1-{\alpha }_{p})}^{4.65}$$

The terminal velocity of particles in the laminar flow regime (certainly the case for group A powders) is given by:

10 $${U}_{t}={[\frac{4g{d}_{sv}({\rho }_{s}-{\rho }_{g})}{3{\rho }_{g}{C}_{D}}]}^{1/2}$$

Since *ρ*_*s*_ – *ρ*_*g*_≈*ρ*_*s*_, the equations can be introduced in Eq. (6) and Eq. (7), resulting in:

11 $${G}_{s}=\frac{{\rm{\Delta }}P}{({U}_{g}-K{U}_{t})+\frac{{K}^{2}gL}{({U}_{g}-K{U}_{t})}}$$

The structure of this transport equation reveals that for G_s_ to be positive, several conditions should be met:

(i) Δ*P* should exceed the sum of all operation-related energy losses.

(ii) *U*_*g*_ should exceed *KU*_*t*_. Although *α*_*p *_≤ 1 and hence *K* < 1, this condition implies that operation of the upflow system cannot be secured at low values of the superficial air velocity, i.e. when *U* ≤ *K U*_*t*_.

(iii) The equation reveals that there will be a maximum in the (G_s_-U_g_) relationship. Setting d*G*_*s*_/*dU*_*g*_ = 0 will determine the location of the maximum and results in *U*_*max*_~ $\sqrt{{K}^{2}gL}$. For *K* = 0.1, *U*_*t*_ = 0.2 m/s and L = 1 m, *U*_*max*_ = 0.33 m/s, which is beyond the terminal velocity implying that the conveying moves into a dilute pneumatic mode.

An additional concern for the potential UBFB applications, such as solar receivers however limits the gas velocities that could be employed since high operating gas velocities are prohibitive. Since gas and particles temperatures will approach equality, the gas will leave the receiver at the discharge (top) particle temperature, hence contributing to sensible heat losses that need to be limited. Particle attrition and tube erosion will moreover be very limited at low superficial gas velocities^41^.

### Efficiency of the UBFB conveyor system

The efficiency of the UBFB conveyor system can be calculated by comparing the compression and uplift work.

The total gas flow rate, F_T_, through the system is determined by ${U}_{g}\cdot \frac{\pi {D}^{2}}{4}$, in m^3^/s. This total gas velocity is the sum of the operating velocity of the dispenser (~1.2 *U*_*mb*_) and the superficial gas velocity induced by the secondary gas injection in the tube.

To pressurize this air flow to the required pressure of the operating ΔP (in kPa) above the atmospheric pressure (101 kPa) work is needed in the compressor, *W*_*s*_, as:

12 $${W}_{s}=\frac{k}{k-1}(101){F}_{T}[{(\frac{101+{\rm{\Delta }}P}{101})}^{\frac{k-1}{k}}]\frac{1}{{\eta }_{c}},\,(in\,kW)$$

With η_c_ the mechanical efficiency of the air mover (i.e. 0.55–0.75 for a turboblower; 0.6–0.8 for a Roots blower and 0.8–0.9 for an axial blower or reciprocating compressor).

The work, *W*_*r*_, required to lift the powder for a height L is

13 $${W}_{r}={G}_{s}\frac{\pi {D}^{2}}{4}L\frac{g}{{10}^{3}},\,(in\,kW)$$

The efficiency of the upward conveying, η, is hence

14 $${\rm{\eta }}=\frac{{W}_{r}}{{W}_{s}}\times 100,\,\text{in}\, \% $$

## Discussion

### Conveying parameters

Results show that there is no apparent effect of particle size and density for both group A powders. *G*_*s*_ increases linearly with U. This is expected from Eq. (15): since $\tfrac{{K}^{2}gL}{U-{U}_{t}}\gg (U-K{U}_{t})$ under the operating conditions, the equation can be simplified to

15 $${G}_{s}=\frac{{\rm{\Delta }}P(U-{U}_{t})}{{K}^{2}gL}$$

**Figure 5:**
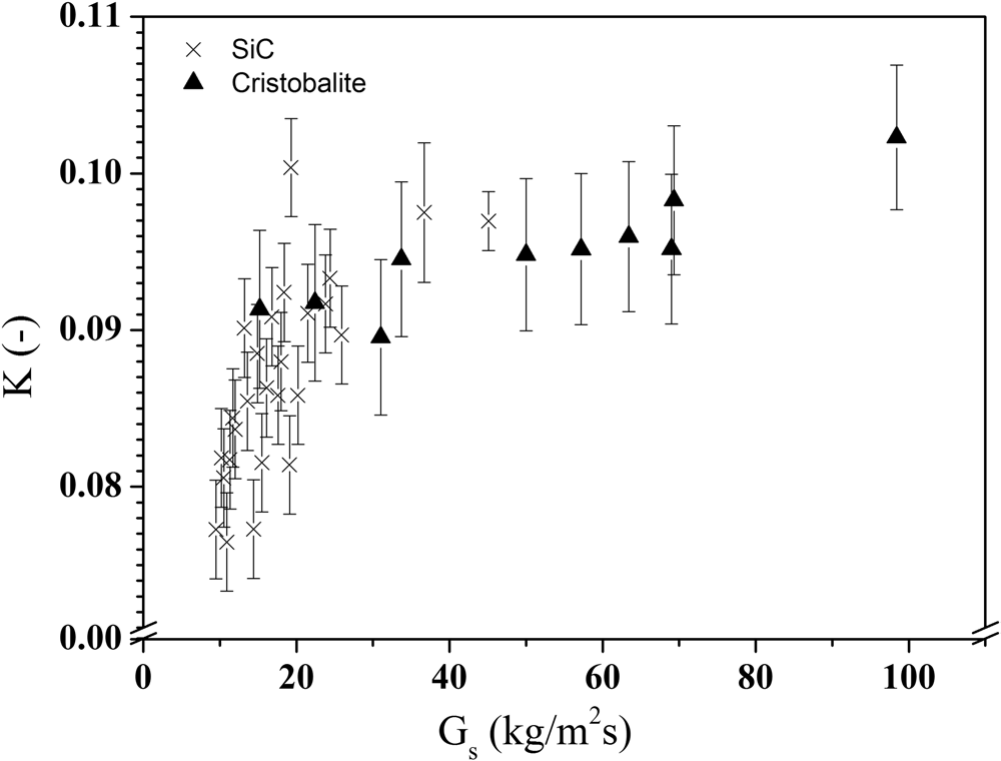
K-values fitted from experimental results.

The parameter *K* was determined from the experimental data, and is represented in Fig. 5.

At higher G_s_-values, *K* is ~0.1, which corresponds to an *α*_*p*_ value of 0.38 (ε = 0.62) according to Eq. (9). Experimentally determined α_p_– values were slightly lower. It should moreover be remembered that α_p_ will increase at low U and could reach its value at *U*_*mf*_, considered about 10% higher than that in packed bed conditions^2^. The higher limit of α_p_ is hence around 0.45 for cristobalite and SiC. At these α_p_ values, Eq. (15) predicts *K* at ~0.064. The downward trend of *K* at lower *G*_*s*_ is therefore logical, and the average 0.10 should only be used for *G* ≥ 20 kg/m^2^s.

The accuracy of the model can be determined by differentiating Eq. (16) and introducing the relative errors for ΔP, U and K, assuming such to be negligible for g and L.

16 $$\frac{d{G}_{s}}{{G}_{s}}=\frac{d({\rm{\Delta }}P)}{{\rm{\Delta }}P}+\frac{d(U-K{U}_{t})}{(U-{U}_{t})}+\frac{2dK}{K}$$

With errors on measured parameters of maximum 2% for ΔP, 5% for K and 5% on U, the total relative error is 12%, recognised as a fair percentage. It should moreover be remembered that the superficial air velocity is determined by its mass flow rate measurement, at a pressure corresponding to the pressure at the bottom of the conveying tube, i.e. (101 kPa + *α*_*p*_ *gρ*_*s*_ *L*). This air will expand towards the disengagement vessel at atmosphere pressure. Although this is of limited effect for conveying tubes of smaller length, the expansion must be taken into consideration for taller conveying tubes. The gradual pressure reduction as the gas moves up the tube will induce an expansion of the gas flow rate, proportional with the reducing pressure at each height. For a given *G*_*s*_-value, the *α*_*p*_ value will decrease, and so will *K*, thus reducing the impact of hindered settling and facilitating the upward solids flow. A similar effect of gas expansion will occur if the upward conveyor is operated at higher temperatures, such as in solar receivers. The particle terminal velocity*, U*_*t*_, decreases with increasing temperature as a result of the increasing gas viscosity, and the gas expansion (with higher effective superficial gas velocity as a result) will again enhance the transport of the solids. Both the effect of tube length and operating temperature must be experimentally verified, and related research is ongoing.

**Figure 6:**
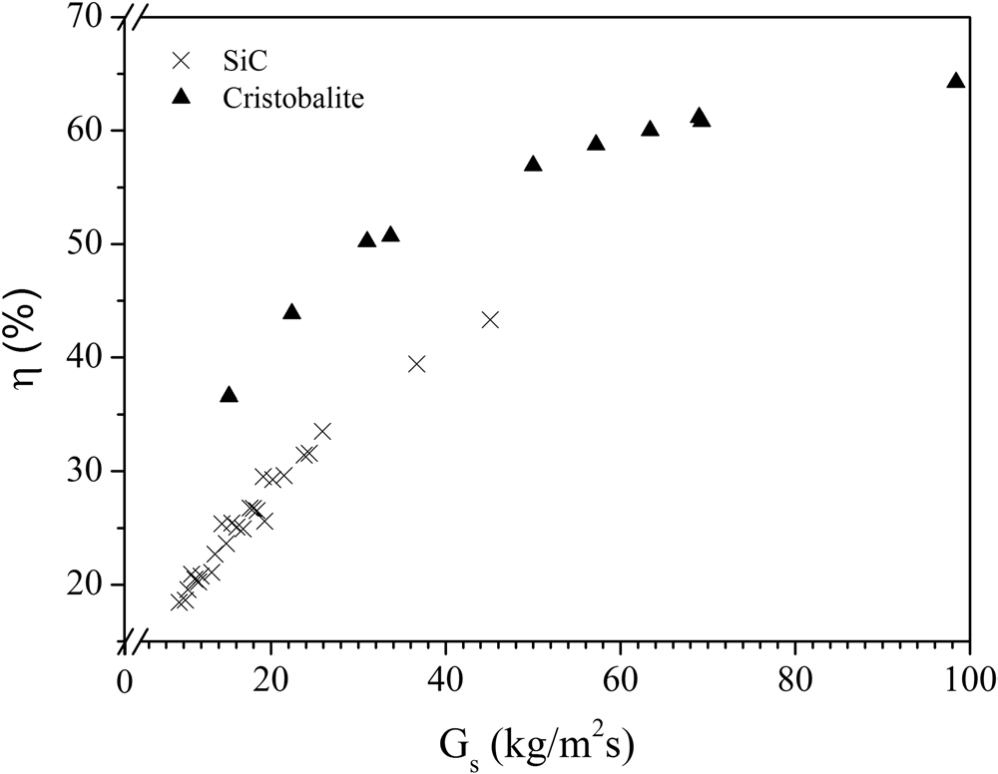
Calculated efficiency of the UBFB conveying.

Figure 6 illustrates the transport efficiency obtained for both powders. The maximum efficiency of the UBFB conveying is assumed ~50% for SiC and 65% for cristobalite. The higher efficiency for cristobalite at equal air velocities is due to the lower operating pressure (9000 Pa instead of 26500 Pa for SiC) as a result of the shorter tube (1 instead of 2.1 m) and lower ρ_s_ (2340 instead of 3210 kg/m^3^).

### Additional considerations

To evaluate the use of the modelling equations, predictions were performed by way of examples and illustrated below. Operating parameters are given in the figure captions.

**Figure 7:**
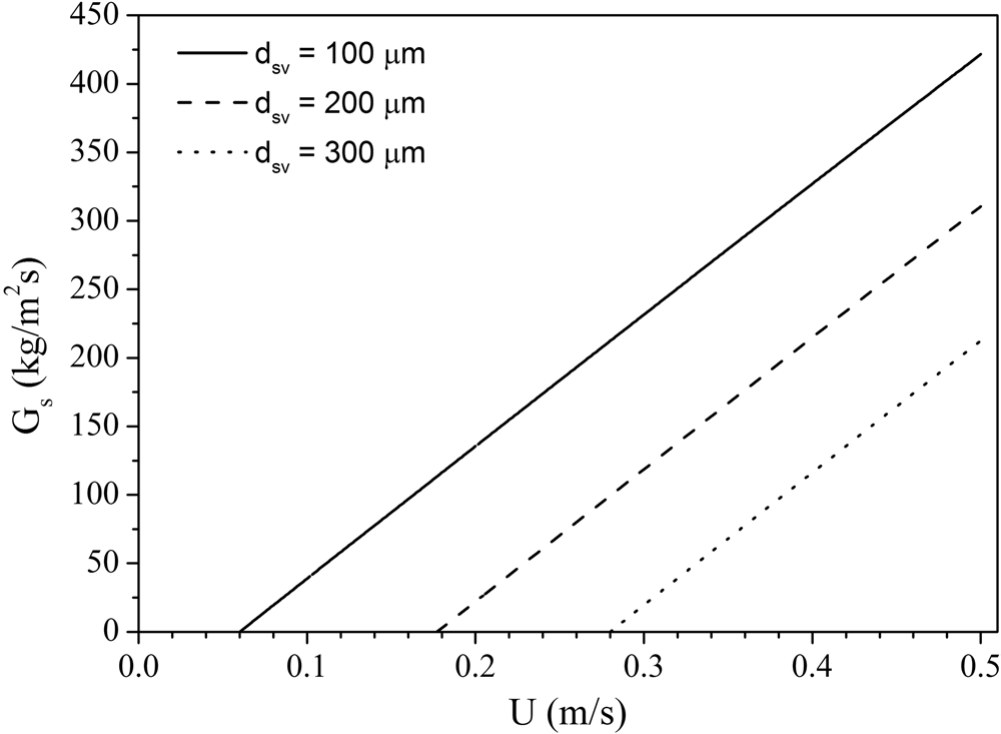
Predicted conveying flux versus air flow rate for cristobalite, ρ_p_ = 2340 kg/m^3^, ψ = 0.67, α_p_ = 0.38, L = 2 m, ΔP = 19200 Pa.

Group B powders still have a fairly low U_t_, K was calculated for α_p_ = 0.4. Predicted (G_s_, U) trends are illustrated in Fig. 7. The increasing onset superficial gas velocity for particles of increasing diameter, is due to their increasing respective terminal velocity. For group D powders, the UBFB transport should be possible but their high U_t_ even at low K will necessitate very high operating U, with excessive attrition and tube erosion as a consequence^41^.

**Figure 8:**
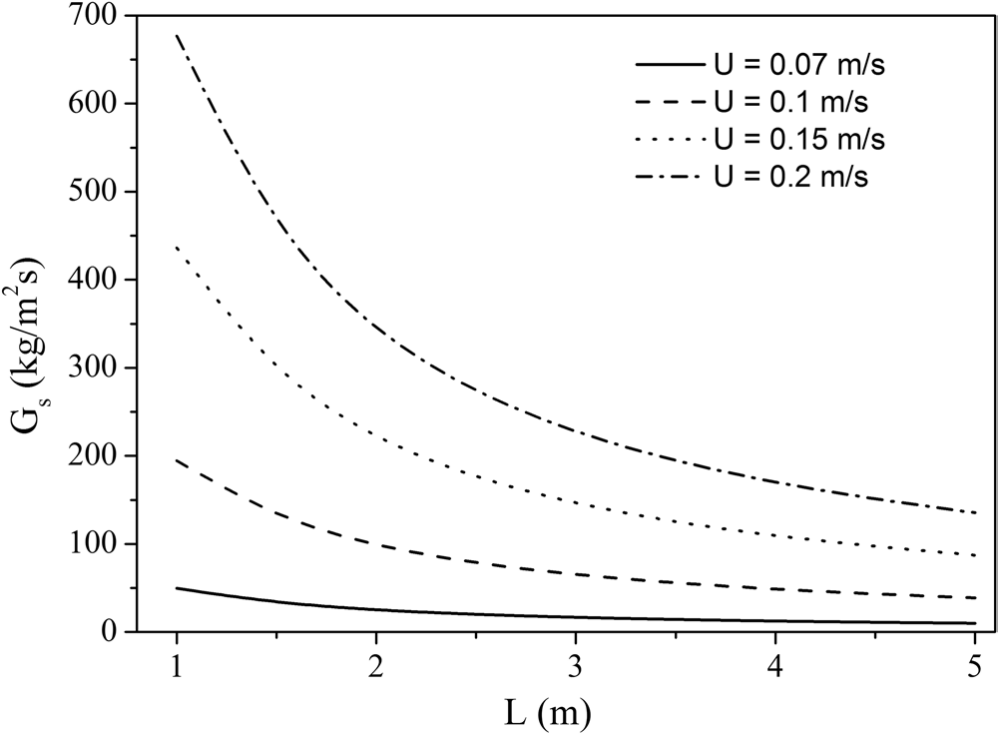
Effect of L at constant externally exerted pressure, UBFB, cristobalite, ρ_p_ = 2340 kg/m^3^, ψ = 0.67, α_p_ = 0.38, Tube length, L, in m.

The effect of the tube length, L, is illustrated in Fig. 8. Tube lengths ≥3m will be applied in the particle-in-tube solar receivers and G_s_-values of 50 to 150 kg/m²s will hence only be achieved by applying a high external pressure and/or a higher superficial velocity.

At a fixed externally exerted pressure, an increasing superficial gas velocity enhances the solid upward flux. For the calculations, the exerted pressure was set at 10% above the hydrostatic pressure drop of the fluidized bed for a given tube length, i.e. ΔP = 1.1 α_p_ ρ_p_ gL (e.g. 48800 Pa for a 5 m long tube, or 9760 Pa for a 1 m long tube).

**Figure 9:**
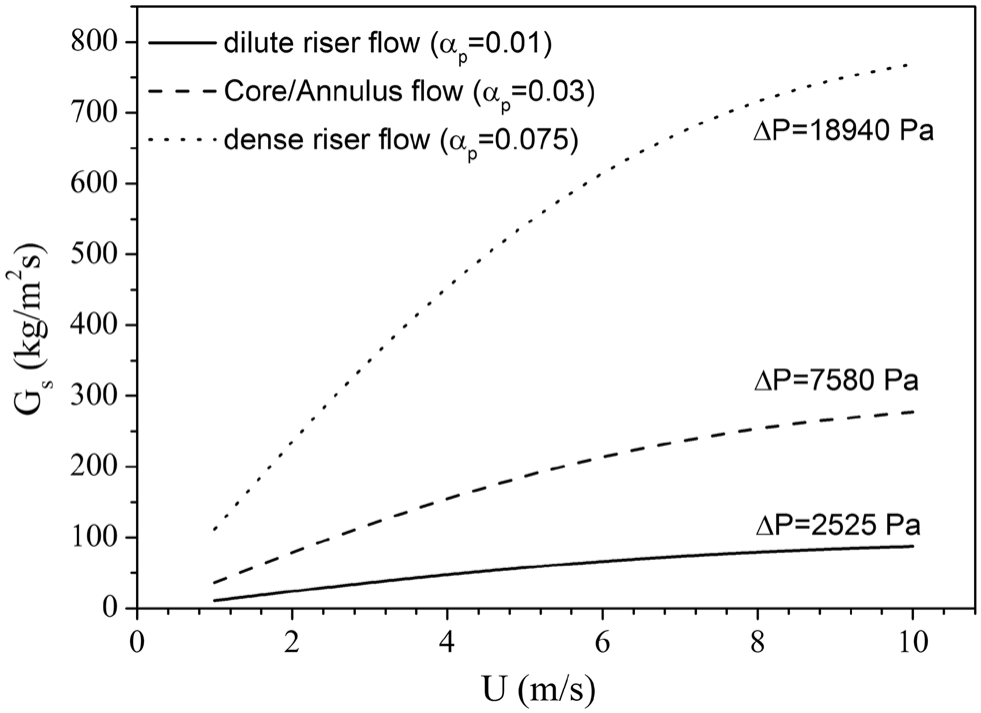
(G_s_, U) trend in a CFB, for cristobalite, ρ_p_ = 2340 kg/m^3^, ψ = 0.67, α_p_ = 0.38.

Finally, the model equations were tentatively applied to common CFB operation modes. This tentative prediction is not directly related to ongoing solar receiver development, but illustrates the potential use of the design equations for CFB operation, as shown in Fig. 9. Values of G_s_ versus U are in-line with normally predicted G_s_-U relationships^42^.

## Conclusions

The upward flow of particles in an Upflow Bubbling Fluidized Bed (UBFB) is studied experimentally for group A powders. For such, the upward solid flux in the tube is proportional with both the applied superficial gas flow rate and the pressure drop exerted at the base of the conveyor tube, and inversely proportional with the tube length. Results are modelled from pressure drop considerations and energy loss equations. The model expression ${G}_{s}=\tfrac{({\rm{\Delta }}P)}{({U}_{g}-K{U}_{t})+\tfrac{({K}^{2}gL)}{({U}_{g}-K{U}_{t})}}$ can be used for design purposes, with K, the correction factor for hindered settling of the particles, equal to 0.1 for the group A powders tested. The energy efficiency of the system increases with increasing U and G_s_, and decreasing particle size and/or density. For SiC and cristobalite, the air velocity required to transport up to 100 kg/m²s of powder was below 0.15 m/s at an inlet pressure slightly exceeding the hydrostatic bed pressure. The model equation was tentatively applied to predict the effects of particle size, tube length and operation in Circulating Fluidized Bed mode. Although predictions seem fair and in-line with expectations, the model expression should however be used with caution for other tube geometries and/or operating modes. It is however demonstrated that the UBFB is an efficient and flexible way of transporting particles upwards, with limited particle attrition and tube erosion due to the low gas and solids velocities applied.

## Electronic supplementary material

Supplementary Information
