# Supplementary material for: Experiments support an improved model for particle transport in fluidized beds
Source: Sci Rep. 2017 Aug 31;7:10178. doi: 10.1038/s41598-017-10597-3 (PMC5579236; doi:10.1038/s41598-017-10597-3)
Supplement: Supplementary file 1 — Supplementary Information [file 41598_2017_10597_MOESM1_ESM.doc]

**Experiments support an improved model for particle transport in fluidized beds**

Huili Zhang^1^, Weibin Kong^1^, Tianwei Tan^1^, Flamant Gilles^2^, Jan Baeyens^1,3^*

**Supplementary material**

**Table S1**. Characteristics of common particle-in-tube conveying systems

| Conveying System | | Common U- range (m/s) | Solid/Gas ratio (kg/kg) | Powder groups commonly conveyed |
| --- | --- | --- | --- | --- |
| Pneumatic | Dilute phase | >U_CH_ or >U_salt_ | 1-5 | A, B, D, (C) |
|  | Dense phase (pulsed, by-pass) | 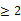 | 100-250 | A, B, C |
| Circulating Fluidized Bed (CFB) | Dilute | >U_TR_ | 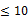 | A, near -B |
|  | Core-annulus |  | 10-100 |  |
|  | Dense |  | >>100 |  |
| Upflow Bubbling Fluidized Bed (UBFB) | | 0.03 – 0.2 | 50-2000 | A, B |

Equations for the choking velocity, U_CH_ (vertical conveying), saltation velocity, U_salt_ (horizontal conveying) and the velocity of transition to the CFB operating mode, U_TR_ have been summarized by Zhang et al.^1^

**Table S2**. Major advantages and drawbacks of the conveying systems

| System | Advantages | Drawbacks |
| --- | --- | --- |
| Pneumatic | -horizontal or vertical conveying  -all powder groups  -commonly single gas injection in pre-mixing chamber  -low pressure drop in dilute conveying | -high pressure drop in dense conveying  -high gas flow rate 🡪 expensive if inert gas is needed  🡪particle separation and gas de-dusting required  🡪 particle attrition and tube erosion |
| CFB | -fairly unlimited column diameter and height  -high solid fluxes possible in the dense operation mode | -vertical conveying only  -limited to group A and near -B powders  -moderate (dilute) to high (dense) pressure drop  -moderate to very high gas flow rate (see above) |
| UBFB | -group A, B, D powders can be used (also moderately cohesive powders)  -very low gas velocity 🡪 negligible attrition and erosion  -use of a feeding fluidized bed puts powder in movement and facilitates solid flow  -secondary air injection stabilizes the transport | -vertical conveying only  -moderate to high pressure drop  -pressurization of dispenser feeding bed required |

**Table S3**. Literature review on group A particles’ fluidized upflow systems

| Reference | Powder, d_sv_(µm)-ρ_p_(kg/m^3^) | I.D. (m) | L_T_(m) | Components of the system | Conveying mode | ΔP/H  (mbar/m) |
| --- | --- | --- | --- | --- | --- | --- |
| Tomita et al. ^2^ | Cement, 30-2560 | 0.41/0.668 | 24.0 | CFB with slow bed, downcomer and L-valve | M to D | 53.8-115.8 |
| Li and Kwauk ^3^ | Resin, 65-1188 | 0.047 | 8.0 | pressurized feeding hopper, rotary valves, UBFB, disengagement, cyclone, intermediary storage hopper | L to M | 125-188 |
| Hirama et al. ^4^ | HA54, 38-750  FCC-catalyst, 57-930 | 0.10  0.10 | 5.5 | pressurized feeding hopper, UBFB, disengagement, L-valve | L to M | 2-180  5-150 |
| Zhu and Zhu ^5^ | FCC-catalyst,65-1780 | 0.101 | 3.6 | pressurized feeding hopper, screw feeder, UBFB, cyclone, intermediary storage hopper | M | 45-55 |
| Turzo ^6^ | Alumina, 54-2360 | 0.028 | 6.0 | pressurized feeding hopper, rotary valves, UBFB, secondary air injection, disengagement | M to VD | 80 |
| Flamant et al ^7^ | SiC, 64-3120 | 0.036 | 2.0 | pressurized feeding hopper, rotary valves, UBFB, secondary air injection, disengagement | M to VD | -120 to 160 |
| Pitie et al. ^8^ | Sand, 75-2260 | 0.05 | 2.5 | CFB with slow bed, downcomer, and L-valve | M to VD | 3.5-5 |
| Zhang et al. ^9^ | Sand, bituminous coal, 74 to 79-2160 to 2340 | 0.05 | 2.5 | CFB with slow bed, downcomer, and L-valve | L to M | 2.1-2.3 |
| Zhang et al. ^10^ | SiC, 64-3120 | 0.029 | 1 | pressurized feeding hopper, rotary valves, UBFB, secondary air injection, disengagement | M to VD | 110-125 |
| Zhang et al. ^11^ | Cristobalite, 58-2340 | 0.05  0.046 | 0.5 | pressurized feeding hopper, rotary valves, UBFB, secondary air injection, disengagement | M to VD | 70-90 |

Mode: *G_s_/G_g_*<20, lean (L); 20 <G_s_/G_g_<100, moderate (M); G_s_/G_g_
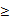
100, dense (D); G_s_/G_g_
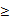
1000, very dense (VD).

**References:**

1. Zhang, H. L., Degrève, J., Baeyens, J. & Dewil, R. Choking affects the operation diagram of a CFB riser. *J. Powder Technol.* **2014,** (2014).

2. Tomita, Y., Yutani, S. & Jotaki, T. Pressure drop in vertical pneumatic transport lines of powdery material at high solids loading. *Powder Technol.* **25,** 101–107 (1980).

3. Li, H. & Kwauk, M. Vertical pneumatic moving-bed transport—II. Experimental findings. *Chem. Eng. Sci.* **44,** 261–271 (1989).

4. Hirama, T., Takeuchi, H. & Chiba, T. Regime classification of macroscopic gas—solid flow in a circulating fluidized bed riser. *Powder Technol.* **70,** 215–222 (1992).

5. Zhu, H. & Zhu, J. Gas-solids flow structures in a novel circulating-turbulent fluidized bed. *AIChE J.* **54,** 1213–1223 (2008).

6. Turzo, G. Transport par fluidisation en phase hyperdense: amélioration technologique. *INPT* (2013).

7. Flamant, G. *et al.* Dense suspension of solid particles as a new heat transfer fluid for concentrated solar thermal plants: On-sun proof of concept. *Chem. Eng. Sci.* **102,** 567–576 (2013).

8. Pitie, F., Zhao, C. Y., Baeyens, J., Degrève, J. & Zhang, H. L. Circulating fluidized bed heat recovery/storage and its potential to use coated phase-change-material (PCM) particles. *Appl. Energy* **109,** (2013).

9. Zhang, H. L., Baeyens, J., Degrève, J., Brems, A. & Dewil, R. The convection heat transfer coefficient in a Circulating Fluidized Bed (CFB). *Adv. Powder Technol.* **25,** 710–715 (2014).

10. Zhang, H. L. *et al.* Particle circulation loops in solar energy capture and storage: Gas–solid flow and heat transfer considerations. *Appl. Energy* **161,** 206–224 (2016).

11. Zhang, H. *et al.* High-efficency solar power towers using particle suspensions as heat carrier and thermal energy storage. *Renew. Energy* **111,** 438–446 (2017).
